# Anti-Platelet Activity of Sea Buckthorn Seeds and Its Relationship with Thermal Processing

**DOI:** 10.3390/foods13152400

**Published:** 2024-07-29

**Authors:** Natalia Sławińska, Jerzy Żuchowski, Anna Stochmal, Beata Olas

**Affiliations:** 1 Department of General Biochemistry, Faculty of Biology and Environmental Protection, University of Lodz, 90-236 Lodz, Poland; beata.olas@biol.uni.lodz.pl; 2 Department of Biochemistry and Crop Quality, Institute of Soil Science and Plant Cultivation—State Research Institute, Czartoryskich 8, 24-100 Pulawy, Poland; jzuchowski@iung.pulawy.pl (J.Ż.); anna.stochmal@gmail.com (A.S.)

**Keywords:** blood platelet activation, hemostasis, *Hippophae rhamnoides* L., roasting

## Abstract

Sea buckthorn (*Hippophae rhamnoides* L.) is a tree or shrub with small, orange berries. Sea buckthorn seeds have shown many properties beneficial to human health, including antioxidant, anti-hypertensive, anti-hyperlipidemic, and retinoprotective activities. Seeds, as a component of food, are often exposed to high temperatures, which can increase or decrease their biological activity. In our previous study, we showed that both raw and roasted sea buckthorn seeds had significant antioxidant activity, which was measured in human plasma in vitro. In this paper, we evaluated the effect of extracts from raw and roasted sea buckthorn seeds on several parameters of hemostasis in vitro, including thrombus formation in full blood (measured by the Total Thrombus formation Analysis System—T-TAS), blood platelet activation (based on the exposition of P-selectin, the active form of GPIIb/IIIa on their surface and platelet-derived microparticles formation), aggregation (measured with impedance aggregometry), adhesion to fibrinogen and collagen, arachidonic acid metabolism in washed platelets stimulated by thrombin, and COX-1 activity. We also measured the levels of free 8-isoprostane in plasma and the total non-enzymatic antioxidant status of plasma. The extract from roasted seeds (50 µg/mL) significantly prolonged the time of occlusion measured by T-TAS—the AUC_10_ (area under the curve) value was decreased by approximately 18%. Both extracts decreased the exposition of the active form of GPIIb/IIIa on the surface of platelets activated with 10 μM ADP (by 38.4–62.2%) and 20 μM ADP (by 39.7–51.3%). Moreover, the extract from raw seeds decreased the exposition of P-selectin on the surface of platelets stimulated with 20 μM ADP (by 31.2–34.9%). The adhesion of thrombin-stimulated platelets to fibrinogen and collagen was inhibited only by the extract from roasted sea buckthorn seeds (by 20–30%). Moreover, the extract from raw seeds inhibited the level of TBARS (thiobarbituric acid-reactive substances, an indicator of enzymatic peroxidation of arachidonic acid) in washed platelets stimulated with thrombin; the activity of COX-1 was inhibited by both extracts, although the effect of the extract from raw seeds was stronger. These results indicate that sea buckthorn seeds have anti-platelet activity that is not decreased by thermal processing, but more research is needed to determine which exact chemical compounds and mechanisms are responsible for this phenomenon.

## 1. Introduction

Sea buckthorn (*Hippophae rhamnoides* L.) is a tree or shrub with small, orange berries. It originated from sandy or mountainous areas of Europe and Asia, where it has been known for its medicinal properties for thousands of years [1,2]. Nowadays, researchers have found many beneficial effects of sea buckthorn fruits, pulp oil, and seed oil, but far less is known about its leaves, twigs, bark, and the seeds themselves [1,2,3,4]. As seeds contain only 12–13% oil, there is still much to be discovered about their content and activity [3]. Moreover, seeds are often seen as waste by the food industry and are discarded in the process of juice, jam, or oil production, even though they contain large amounts of beneficial compounds that could be extracted and reclaimed [4].

The global sea buckthorn market is estimated to be worth USD 347.56 million in 2023 and is expected to continue growing. The Asia Pacific region has the biggest share, which is estimated to be 67.52% [5]. Various products, including food, dietary supplements, pharmaceuticals, and cosmetics are available on the market; the majority is made from sea buckthorn fruits. Notably, multiple sea buckthorn products used to treat ischemic cardiopathy are available in China [5,6,7].

Anti-platelet drugs have an important function in the prevention and treatment of cardiovascular diseases (CVDs) but can also induce various side effects. Patients suffering from CVDs, like ischemic heart disease, often must take anti-platelet drugs such as aspirin and its derivatives. Unfortunately, the prolonged use of this type of drugs can cause bleeding from the gastrointestinal tract. For this reason, researchers continue to search for new anti-clotting (anti-platelet) compounds that could be used in pharmacotherapy [6,8]. Certain dietary components such as phenolic compounds and supplements with anti-platelet activity can inhibit platelet activation and might play a role in the prophylaxis and treatment of CVDs. Studies have shown that polyphenols can affect platelet activation, degranulation, aggregation, and adhesion by targeting different thrombogenic pathways, for example the COX-1-thromboxane pathway, and thrombin- or collagen-induced platelet activation [9].

Oxidative stress is implicated in many diseases, including ischemic heart disease [10,11]. Several studies have already demonstrated the antioxidant properties of sea buckthorn seeds [2,4,12,13,14], but the effect of thermal processing on their biological activity has not been studied yet. Seeds, as a component of food, are often exposed to high temperatures, for example during bread baking or oil extraction; seeds can also be roasted before consumption to improve their flavor and color or boiled to increase digestibility. The temperatures of processing can vary greatly; for example, to make rye bread, the temperature is first brought up to 280 °C (for ~10 min) and then reduced to 175 °C, while oil extraction can be performed at both low (cold pressing) and high temperatures (during the solvent extraction of canola oil, the temperature can exceed 200 °C) [12,15]. Numerous studies have shown that high temperatures can increase or decrease the strength of different biological activities. For this reason, thermal stability is an important factor to consider while studying plant-derived compounds [16,17]. Our preliminary data showed that high temperature does not impair the antioxidant activity of sea buckthorn seeds [12]. Although there have been reports of antithrombotic properties of other parts of sea buckthorn [18], the effect of raw (no thermal processing) and roasted (thermally processed) sea buckthorn seeds on hemostasis has not been studied yet. However, our preliminary results have shown that the extract from the roasted seeds (at the concentration of 50 µg/mL) prolonged the time needed for white thrombus formation, which was measured with the Total Thrombus-formation Analysis System (T-TAS) AR chip [12]. Therefore, the aim of this study was to determine the anti-platelet activity of sea buckthorn seeds and its relationship with thermal processing in vitro. In continuation of our previous research, we used the T-TAS PL chip to assess the effects of the extracts from raw and roasted sea buckthorn seeds on primary hemostasis, including the blood platelet thrombus formation process, which involves blood platelet adhesion, aggregation, granule secretion, and thrombus growth. Moreover, flow cytometry was used to determine if the tested extracts had anti-platelet potential on the basis of platelet microparticles formation and the exposition of P-selectin and the active form of GPIIb/IIIa on the surface of resting or agonist-stimulated blood platelets in vitro. We also evaluated the anti-aggregatory and anti-adhesive properties of the extracts. Additionally, we investigated the effect of the extracts on various biomarkers of oxidative stress in vitro: the level of free 8-isoprostane in plasma, the total non-enzymatic antioxidant status of plasma, and enzymatic lipid peroxidation—arachidonic acid metabolism in platelets activated by thrombin. As arachidonic acid can be metabolized by cyclooxygenase (COX), we also studied the effect of raw and roasted sea buckthorn seeds on COX-1 activity. Moreover, we compared the activity of the tested extracts with the activity of a commercial product, Aronox (*Aronia melanocarpa* berry extract with anti-platelet and antioxidant properties) [18,19].

## 2. Materials and Methods

### 2.1. Chemicals

Methanol, hexane, and butanol used to prepare sea buckthorn seed extracts were obtained from Merck (Darmstadt, Germany). Antibodies (CD62P/PE, PAC-1/FITC, CD61/PerCP, and CD61/PE) were acquired from Becton Dickinson (Franklin Lakes, NJ, USA). PL chips and other equipment needed for T-TAS were purchased from Bionicum Sp. z o.o. (Warsaw, Poland). The kits for measuring 8-isoprostane level, total non-enzymatic antioxidant status, and COX-1 activity were from Cayman Chemical (Ann Arbor, MI, USA). A stock solution of Aronox (*A. melanocarpa* berry extract) was purchased from Agropharm Ltd. (Warsaw, Poland). Phosphate-buffered saline (PBS), tris(hydroxymethyl)aminomethane (Tris), fibrinogen, bovine serum albumin (BSA), 4-nitrophenyl phosphate, adenosine diphosphate (ADP), collagen, fibrinogen, and thrombin were from Merck (Darmstadt, Germany). All the other reagents were purchased from commercial suppliers, including POCH (Gliwice, Poland) and Chempur (Piekary Śląskie, Poland).

### 2.2. Plant Material

The plant material is also described in our previous work [12]. Whole branches of sea buckthorn were supplied by a horticultural farm in Sokółka, Podlaskie Voivodship, Poland (53°24′ N, 23°30′ E). The fruit was picked by hand and stored in a freezer. The thawed fruit was roughly homogenized in water with a blender. The seeds sedimented, skins and pulp were washed out with water. The seeds were drained, air-dried, and stored at room temperature. To check if sea buckthorn seeds could be used as an addition to bread without losing their biological activity, thermal treatment was applied to a portion of the seeds. From an organoleptic point of view, the addition of seeds to the bread mass has a positive effect on the taste of the bread. A portion of the seeds was subjected to thermal treatment in a baking company. The total roasting time was 36 min, and the initial temperature in the baking chamber was 280 °C (for ~10 min.), which was subsequently reduced to 175 °C [12]. All procedures complied with relevant institutional, national, and international guidelines and legislation.

### 2.3. Preparation of the Extracts from Raw and Roasted Sea Buckthorn Seeds

Extracts from the raw and roasted sea buckthorn seeds were prepared according to procedures described in detail in our earlier Open Access publication [12]. Briefly, powdered seeds (both variants) were defatted with hexane. The raw seed powder (282.4 g) was extracted with 2.5 L of 80% methanol (*v*/*v*), for 5 h at room temperature, and then with 2.5 L of methanol, under the same conditions. After filtration, the extract was defatted by liquid–liquid extraction with hexane and rotary-evaporated to remove organic solvents. The defatted crude extract was rotary-evaporated to remove methanol and subjected to liquid–liquid extraction with *n*-butanol. The obtained butanol fractions were dried in a rotary evaporator, dissolved in 20% *tert*-butanol in MilliQ water, and freeze-dried (Gamma 2-16 LSC, Christ, Osterode am Harz, Germany). Finally, 1.566 g of the raw seed extract was obtained. The powdered roasted seeds (91 g) were subjected to ultrasound-assisted extraction in an ultrasonic bath: 500 mL 80% methanol (*v*/*v*; 30 min), 400 mL 80% methanol (30 min), 300 mL 80% methanol (30 min), and 200 mL 100% methanol (20 min). The next steps of the extract preparation were the same as those described above and yielded 1.930 g of the roasted seed extract. The extracts were stored in a sealed falcon tube, in the dark, at 4 °C.

### 2.4. Preparation of Stock Solutions of the Extracts for Bioassays

The extracts were dissolved in 50% DMSO (a universal solvent for many different plant substances) and stored at −20 °C. The final concentration of DMSO in the blood samples was below 0.05% (*v*/*v*). It is important to note that the addition of a low concentration of DMSO to human blood has no effect on platelet activation and other studied biological properties of hemostasis elements (data not presented). Aronox was dissolved in water. The final concentrations of the extracts in the samples ranged from 0.5 to 50 μg/mL.

### 2.5. Blood Samples and Isolation of Blood Platelets

Fresh human blood was collected at a “Diagnostyka” blood collection point at Brzechwy 7a St., Lodz, Poland. All donors (aged 25–28) were healthy volunteers; none were smokers or reported taking drugs, including anti-platelet, anticoagulant, and antioxidant medication for 14 days prior to blood collection. The blood for flow cytometry assays, platelet isolation, and plasma isolation was drawn into tubes with CPDA anticoagulant (citrate/phosphate/dextrose/adenine; 8.5:1; *v*/*v*; blood/CPDA), while the blood for T-TAS was drawn into tubes with benzylsulfonyl-D-Arg-Pro-4-amidinobenzylamide (BAPA). The analysis of blood samples was performed according to the guidelines of the Helsinki Declaration for Human Research. The blood used in T-TAS or flow cytometry assays was incubated (30 min, at 37 °C) with two extracts at final concentrations of 0.5–50 μg/mL. The experiments were conducted with the consent of the Bioethics Committee of the University of Łódź (number 11/KBBN-UŁ/I/2019). All donors signed an informed consent form one day prior to blood collection.

Plasma was obtained by differential centrifugation (2800× *g*, 20 min, at room temperature) (MPW Med. Instruments, Warsaw, Poland). Blood platelets were also isolated from whole blood with differential centrifugation as described previously [20]. The platelet count was approximated with spectrophotometry at 800 nm. Then, the platelets were diluted to the level of 2.0 × 10^8^/mL with Barber’s Buffer (0.14 M NaCl, 0.014 M Tris, 5 mM glucose, pH 7.4).

### 2.6. Total Thrombus-Formation Analysis System (T-TAS) (Using Whole Blood)

Thrombus formation analysis in full blood was carried out with the Total Thrombus formation Analysis System (T-TAS) PL chip (T-TAS 01, Fujimori Kogyo Co., Ltd, Tokyo, Japan). T-TAS can measure blood clotting in semi-physiological conditions. In the PL chip, blood is pushed through a tube coated with type I collagen. Blood platelets are activated by collagen and 1500/s shear stress. Newly formed thrombi block the flow path, which increases the pressure within the tube. The pressure is recorded and expressed AUC_10_ (area under the curve)—area under the flow pressure curve recorded for 10 min after the test starts. The AUC_10_ parameter provides information about the growth, stability, and intensity of thrombus formation. Blood for the PL chip is drawn into tubes with BAPA anticoagulant, which blocks secondary hemostasis, so only primary hemostasis can be studied. Further information about this method can be found in [21]. The extracts were incubated with full blood at 37 °C for 30 min. The final concentrations were 0.5, 5, and 50 μg/mL. A control sample with 0.9% NaCl instead of the extracts was set up. After the incubation, the samples were gently mixed and placed in the PL chips. The pressure was recorded for 10 min or until the time of occlusion (when the pressure reached 60 kPa). The data were expressed as % of the control sample.

### 2.7. Activation of Blood Platelets Measured with Flow Cytometry (Using Whole Blood)

The effect of the extracts on the activation of blood platelets in full blood was measured by flow cytometry. Two methods were used to assess platelet activation. The first method was based on the exposition of P-selectin (CD62P) and the active form of GPIIb/IIIa (PAC-1) on the surface of platelets. First, the extracts were added to full blood. The final concentrations of the extracts in the samples were 0.5, 1, 5, 10, and 50 μg/mL. A control sample with 0.9% NaCl was set up. The samples were incubated at 37 °C for 15 min. The samples were divided into four experimental groups: unstimulated platelets and platelets stimulated with 10 mM ADP, 20 mM ADP, and 10 μg/mL collagen. Agonists were added to the samples. All samples were incubated again at 37 °C for 15 min. Afterward, the samples were diluted 10× with sterile PBS. Then, 10 μL of diluted samples was placed into new tubes and stained for 30 min in the dark (room temperature) with 3 μL of each CD61/PerCP (cat. no. 347408), CD62P/PE (cat. no. 348107), and PAC-1/FITC (cat. no. 340507) antibodies. Lastly, the samples were fixed with 1% CellFix (37 °C, 1 h). The samples were analyzed with LSR II Flow Cytometer (Becton Dickinson, San Diego, CA, USA). At least 5000 CD61/PerCP-positive objects were recorded. Blood platelets were gated based on a forward light scatter (FCS) vs. side light scatter (SSC) plot on a log/log scale (first gate) and positive staining with CD61/PerCP antibodies (second gate). The percentages of CD62/PE- and PAC-1/FITC-positive platelets were calculated in each sample.

The second method was based on the measurement of platelet-derived microparticles (PMP) formation. First, full blood was incubated with the extracts at final concentrations of 0.5, 5, and 50 μg/mL (15 min, 37 °C). A control sample with 0.9% NaCl was set up. After 15 min, 20 μg/mL collagen was added to half of the samples, while the other half was not stimulated. The samples were incubated for 15 more minutes at 37 °C. Afterward, the samples were diluted 10× with sterile PBS. Then, 10 μL of diluted samples was stained in the dark (room temperature, 30 min) with 2 μL of CD-61/PE (cat. no. 555754) antibody. Lastly, 200 μL of 1% CellFix was added, and the samples were incubated at 37 °C for 1 h. The samples were analyzed with LSR II Flow Cytometer (Becton Dickinson, San Diego, CA, USA). At least 10,000 CD61-positive objects were recorded. PMP were gated based on positive staining with CD61/PE antibody (first gate) and size (second gate).

### 2.8. Blood Platelet Aggregation (Using Whole Blood)

Blood platelet aggregation was measured with whole blood impedance aggregometry with the help of a Multiplate Analyzer (Roche, Basel, Switzerland). First, blood samples were incubated with the extracts at 37 °C for 30 min (final concentrations: 5 and 50 μg/mL). A control sample with 0.9% NaCl was set up. Then, 300 μL of pre-heated (37 °C) NaCl/CaCl_2_ solution (Roche) and 300 μL of the samples were added to cuvettes and incubated for 3 min at 37 °C. The samples were stirred continuously throughout the duration of the test. After the incubation, 20 μL of 0.2 mM ADP was added, and the aggregation was measured for 6 min. The results were recorded as AUC (area under the curve) and U (1 U = 10 AU*min) parameters.

### 2.9. Blood Platelet Adhesion (Using Washed Blood Platelets)

Blood platelet adhesion was measured with a method involving the activity of acid phosphatase (a platelet exoenzyme), which was based on the assay described by Bellavite et al. (1994) [22]. First, 96-well plates were coated with adhesion proteins. Then, 100 μL of either fibrinogen (100 μg/mL) or collagen (0.04 μg/mL) was added to the wells, after which the plates were covered with parafilm and incubated at 4 °C on an orbital shaker for 24 h. The next day, the plates were washed three times with TBS (pH 7.5), and 200 μL of 1% BSA was added. The 96-well plates were covered with parafilm and incubated at 37 °C for 2 h. In the meantime, sea buckthorn extracts were added to washed blood platelets at the final concentrations of 0.5, 5, and 50 μg/mL and incubated at 37 °C for 30 min. A control sample was set up containing only blood platelets with Barber’s buffer—the adhesion in this sample was considered to be 100%. After the incubation, BSA was removed from the coated wells. The wells were washed three times with TBS (pH 7.5) with 0.1 mM CaCl_2_, 0.1 mM MgCl_2_, and 0.04% Tween 20. Then, 100 μL of samples was added to the wells in triplicate. In one of the plates, the adhesion of unstimulated platelets to collagen was studied; in this plate, 50 μL of TBS (pH 7.5) was added to the wells. In the rest of the plates, 50 μg of platelet agonists was added; 50 μL of thrombin (final concentration—0.2 U/mL) was added to the wells coated with collagen and fibrinogen, and 50 μL of ADP (final concentration—30 μM) was added to the wells coated with fibrinogen. The plates were covered with parafilm and incubated at 37 °C for 1 h. After the incubation, the wells were washed three times with PBS. Next, 150 μL of 0.1 M citrate buffer (pH 5.4) with 5 mM p-nitrophenyl phosphate and 0.1% Triton X-100 was added to the samples, and the plates were incubated at room temperature for 1 h. Afterward, 100 μL of 2 M NaOH was added, and the absorbance was read at 405 nm with a SPECTROstar Nano Microplate Reader (BMG LABTECH, Ortenberg, Germany). The adhesion was calculated as % of the control sample.

### 2.10. Arachidonic Acid Metabolism in Washed Blood Platelets

Arachidonic acid metabolism in platelets stimulated with thrombin was monitored by measuring the level of thiobarbituric acid reactive substances (TBARS). First, washed platelets were incubated with the extracts at final concentrations of 0.5, 5, and 50 μg/mL. A control sample with Barber’s buffer instead of the extracts was set up. Then, the samples were incubated at 37 °C for 30 min. Afterward, thrombin (final concentration—5 U/mL) was added to the tested samples and to the positive control. Then, 0.9% NaCl was added to the negative control instead of thrombin. All samples were incubated at 37 °C for 5 min; then, equal amounts of 15% trichloroacetic acid (TCA) in 0.25 M HCl and 0.37% thiobarbituric acid (TBA) in 0.25 M HCl were added. The samples were placed in a 100 °C thermoblock for 15 min. Then, they were cooled down to room temperature and centrifuged (10,000 rpm, 15 min, 18 °C). The absorbance of the supernatant was measured at 535 nm with a SPECTROstar Nano Microplate Reader (BMG LABTECH, Ortenberg, Germany). TBARS concentration was calculated with a molar extinction coefficient (ε = 156,000 M^−1^cm^−1^) [23].

### 2.11. COX-1 Activity

The effect of the extracts on cyclooxygenase 1 (COX-1) activity was measured with a COX-1 (human) Inhibitor Screening Assay Kit (Item No. 701070, Cayman Chemical). This assay measures prostaglandin F_2_ alpha (PGF_2α_) by stannous chloride reduction in COX-derived prostaglandin H_2_ (PGH_2_), which is produced in the COX reaction. The prostanoid product is then quantified by ELISA. First, the reaction buffer and Eppendorf tubes were pre-heated to 37 °C. To obtain background values, a tube with COX-1 was placed in boiling water for three minutes to inactivate it. Then, 10 μL of inactive COX-1 was added to a tube with 160 μL of reaction buffer, 10 μL of heme, and 10 μL of 50% DMSO (inhibitor vehicle). To obtain 100% initial activity value, 160 μL of reaction buffer, 10 μL of COX-1, 10 μL of heme, and of 50% DMSO were added to two tubes. To obtain the COX-1 inhibitor samples, 160 μL of reaction buffer, 10 μL of COX-1, 10 μL of Heme, and 10 μL of the extracts (final concentrations: 1, 10, 50, and 50 μg/mL) were mixed. All samples were incubated at 37 °C for 10 min or 30 min. After the incubation, the reaction was initiated by adding 10 μL of arachidonic acid. The samples were incubated at 37 °C for 2 min. Then, a saturated stannous chloride solution was added to stop the reaction. The samples were incubated with stannous chloride at 37 °C for 15 min. Next, the background samples were diluted 100× with ELISA Buffer, and the 100% initial activity and COX-1 inhibitor samples were diluted 2000× and 4000×. The standards were prepared according to the manual. The samples were added to the plate at two dilutions, each in duplicate. The blank, total activity, non-specific binding, and maximum binding wells were set up. Other reagents (ELISA Buffer, Tracer, Antiserum) were added to appropriate wells according to the kit manual. The plate was covered and incubated at 4 °C for 18 h. Afterward, the wells were emptied and washed 5 times with wash buffer. Then, 200 μL of Ellman’s reagent was added to each well, and 5 μL of tracer was added to the total activity well. Lastly, the plate was developed (on an orbital shaker, at room temperature in the dark) until the absorbance of maximum binding wells was higher than 0.3 (blank subtracted). The absorbance was read at 412 nm with a SPECTROstar Nano Microplate Reader (BMG LABTECH, Ortenberg, Germany). COX-1 activity was calculated according to the kit manual. The value of the 100% initial activity sample was considered as 100% COX-1 activity, and the percentage of inhibition of the samples containing the extracts was calculated by subtracting inhibitor samples from the 100% initial activity sample, dividing by the 100% initial activity sample, and multiplying by 100.

### 2.12. The Level of Free 8-Isoprostane in Plasma

The level of free 8-isoprostane in human plasma was determined with an 8-Isoprostane Express ELISA Kit (Item No. 516360, Cayman Chemical). Before the measurements, plasma was tested for interference, and the optimal dilution of the samples was determined. The standards, blank, total activity, non-specific binding, and maximum binding wells were set up according to the manual. The extracts were added to human plasma at the final concentrations of 5 and 50 μg/mL. An oxidative stress inducer (H_2_O_2_/Fe^2+^/EDTA) was added to the test samples and positive control. Then, 0.9% NaCl was added to the negative control, and all samples were incubated at 37 °C for 30 min. Afterward, the samples were added to the wells at two dilutions each in duplicate. Other reagents (AChE Tracer and Antiserum) were added to the wells according to the kit manual. The plate was incubated for 2 h at room temperature on an orbital shaker. Afterwards, the plate was developed with Ellman’s reagent on an orbital shaker, in the dark, at room temperature until the absorbance in the maximum binding wells was above 0.3. The absorbance was read with a SPECTROstar Nano Microplate Reader (BMG LABTECH, Ortenberg, Germany) at 412 nm. The concentration of free 8-isoprostane in the samples was calculated with a pre-configured analysis tool provided by the manufacturer.

### 2.13. Total Non-Enzymatic Antioxidant Status of Plasma

The total non-enzymatic antioxidant status was measured with an Antioxidant Assay Kit (Item No. 709001, Cayman Chemical). This assay is based on the ability of antioxidants to inhibit the oxidation of 2,2′-azino-bis(3-ethylbenzothiazoline-6-sulfonic acid) (ABTS) to ABTS^•+^ by metmyoglobin. First, the extracts were incubated with plasma at 37 °C for 30 min. A control sample with 0.9% NaCl was set up. After the incubation, the samples were diluted (1:20) with assay buffer. The standards were set up according to the kit manual. Then, 10 μL of the samples, 10 μL of metmyoglobin, and 150 μL of chromogen were added to the wells in duplicate. Afterwards, 40 μL of hydrogen peroxide was added to the wells. The plate was covered and incubated on a shaker at room temperature for 5 min. The absorbance was read at 405 nm using a SPECTROstar Nano Microplate Reader (BMG LABTECH, Ortenberg, Germany). The calculations were carried out with a pre-set up tool provided by the manufacturer.

### 2.14. Data Analysis

Statistical analysis was carried out with Statistica 13.3 (StatSoft 13.3, TIBCO Software Inc., Palo Alto, CA, USA). Flow cytometry data were analyzed with Floreada.io online software (accessed on 28 July 2023). Data distribution was checked with the Shapiro–Wilk test, and the homogeneity of variance was checked with Levene’s test. If the data had normal distribution and homogenous variance, one-way ANOVA with Tukey’s post hoc test was used to determine if the differences between groups were statistically significant. Otherwise, the Kruskall–Wallis test was used. The results were considered significant at *p* < 0.05. The results were presented as mean ± SD or median with interquartile range.

## 3. Results and Discussion

Edible seeds, which are abundant in bioactive compounds, play a vital role in human nutrition and wellness. The role of fruit seeds in the human diet has increased considerably, especially in Western countries. They are good sources of nutrients, micronutrients, fiber, and proteins, especially amino acids. Seeds can be used as functional food and added to a wide range of dishes to improve their nutritional content and aid with certain health issues, including cardiovascular diseases. Incorporating edible seeds into the daily diet could be an important step toward health improvement, because antioxidants and other bioactive phytochemicals (for example, anti-platelet compounds) found in seeds are beneficial in various diseases, including CVDs. They can be used whole or ground up, in the form of oils, stable emulsions, or microencapsulated powder that can be incorporated into a variety of food formulations [24,25,26,27]. One of the many ways of including seeds in the diet is adding them to bread dough. The idea of using seeds as an addition to bread requires examining the impact of thermal treatment on the content of active compounds beneficial for health protection and the treatment of heart diseases. For this purpose, the seeds were subjected to a process identical to that used in baking bread. Not only sea buckthorn berries, but also its seeds can be processed into various food products. In the continuation of our study on sea buckthorn seeds, the current paper was aimed at estimating their anti-platelet and antioxidant activities related to hemostasis.

The positive action of various plant preparations is very often correlated with an elevated content of phenolic compounds, which can induce different biological effects by free radical scavenging, metal chelation, and binding receptors or other proteins. Polyphenols can also act as anti-platelet agents by inhibiting platelet degranulation, aggregation, and adhesion. For example, polyphenols can inhibit platelet degranulation by scavenging H_2_O_2_ (a substrate involved in the COX-1 pathway), stimulate nitric oxide production (which can inhibit aggregation), or decrease intracellular Ca^2+^ levels. Polyphenols can also directly attenuate platelet activation by interaction with platelet receptors [9]. In our previous study, we determined that the extracts from raw and roasted sea buckthorn seeds contain mostly glycosides of isorhamnetin, quercetin, and kaempferol. Other compounds, like B-type proanthocyanidins, catechin, and triterpenoid saponins were present as well [12]. These results are consistent with other published data. For example, Wei et al. [2] reported that a flavonoid extract from seed residues of *H. rhamnoides* ssp. *sinensis* contains an abundance of glycosylated forms of isorhamnetin, quercetin, and kaempferol, as well as B-type procyanidins and various flavanol compounds, like (-)-epigallocatechin, (-)-gallocatechin, and (-)-catechin. Small amounts of flavanones (e.g., naringenin-7-glucoside), chalcones, isoflavones, and other flavonoids were also identified.

Sea buckthorn seeds have shown many properties beneficial to human health. Wang et al. [28] demonstrated protective effects of sea buckthorn seed proanthocyanidins (50 and 100 mg/kg/day) against visible light-induced retinal degeneration in vivo. In another study, flavonoid extract from sea buckthorn seeds protected against alcohol-induced intestinal barrier dysfunction [2]. In addition, the results of Chand et al. [29] indicate that sea buckthorn seed supplementation (at the rate of 2 and 3 g/kg) improves egg quality and cholesterol in Rhode Island Red x Fayoumi layers. Importantly, several in vivo studies have shown that sea buckthorn seeds have a positive effect on the cardiovascular system. Pang et al. [30] reported that the total flavones from sea buckthorn seed residues (at a dose of 150 mg/kg/day) had anti-hypertensive activity—they reduced systolic blood pressure from 136.15 ± 1.64 mmHg to 113.3 ± 1.82 mmHg and reduced angiotensin II levels in the plasma of sucrose-fed rats [30]. Flavonoid-enriched seed extract (100–300 mg/kg) reduced body weight, adipocyte size, hepatic triglyceride accumulation, and serum triglycerides in mice fed with high-fat diet [31]. Moreover, in a study by Wang et al. [3], the administration of total flavonoids from seed residues (at a dose of 50–150 mg/kg for 12 weeks) reduced serum total cholesterol and LDL cholesterol in high-fat diet fed mice [3]. These results indicate that sea buckthorn seeds could be useful in improving the health of the cardiovascular system; however, there is a lack of data about their effect on hemostasis.

Therefore, to bridge this gap in knowledge, we used several in vitro models to study the anti-platelet activity of sea buckthorn seeds extracts. It is worth noting that in the present study, we used a combination of T-TAS and flow cytometry to study blood platelet activation in full blood, which is a more natural environment than media in which platelets are suspended after isolation [32]. In addition, we examined the anti-adhesive properties of the two used seed extracts using an in vitro model based on washed blood platelets.

### 3.1. Measurement of Thrombus Formation with T-TAS (PL Chip) and Platelet Aggregation

**Figure 1:**
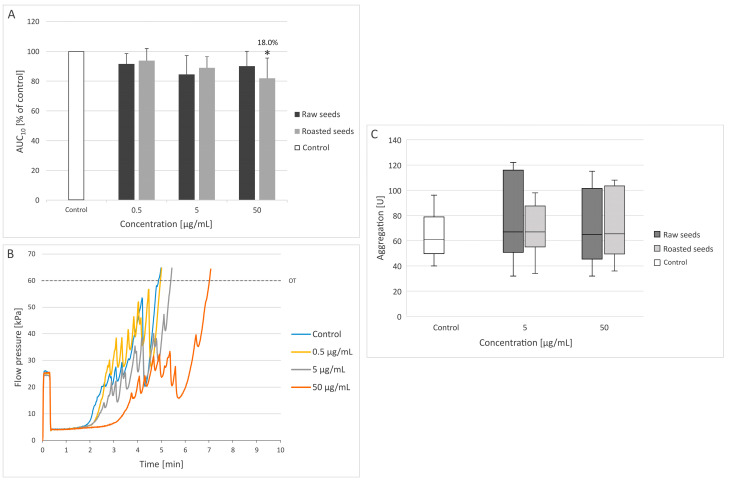
Effect of the extracts from raw and roasted sea buckthorn seeds (at concentrations of 0.5–50 μg/mL) on thrombus formation in whole blood (**A**,**B**) (*n* = 6) and blood platelet aggregation in whole blood (**C**) (*n* = 6). The samples (**A**,**B**) were analyzed with the T-TAS PL chip at the shear stress rates of 1500/s. The results (**A**,**B**) are calculated as AUC_10_ (area under the curve). In the graph (**A**), AUC_10_ is expressed as a percentage of the control sample (blood without the tested extract). The data are expressed as means ± SD (**A**) or median and interquartile range (**C**). The results were considered significant at *p* < 0.05 (* *p* < 0.05). The number above the significant result is the % of inhibition of thrombus formation (**A**). (**B**) demonstrates a selected diagram of the pressure recorded inside the PL chip for 10 min (OT—occlusion time) (for the extract from roasted seeds).

T-TAS is a microchip-based flow chamber system that evaluates thrombogenicity in whole blood and may also be used to assess the effect of anti-thrombotic preparations on platelet activation [33]. For the first time, our results of the T-TAS test (using the PL chip) showed that the AUC_10_ value of the two tested extracts (at concentrations of 0.5, 5, and 50 µg/mL) was reduced compared to the control value (Figure 1A,B). However, only the extract from roasted seeds at the highest concentration (50 µg/mL) demonstrated anti-platelet activity by significantly prolonging the time of occlusion (*p* = 0.0307)—the AUC_10_ value was decreased by approximately 18% (Figure 1A,B). On the other hand, neither of the extracts changed blood platelet aggregation stimulated by ADP, which was measured in whole blood at two concentrations (5 and 50 µg/mL) (Figure 1C).

The T-TAS PL chip evaluates primary hemostasis, i.e., the overall thrombus formation process, including platelet aggregation and adhesion, granule secretion, and thrombus growth. It is approved for use in clinical laboratories and has been successfully utilized to monitor the effectiveness of individual and combined anti-platelet drugs in cardiovascular patients [33,34,35]. The thrombus formation process in the PL chip takes place on the surface of collagen, with the involvement of release of platelet agonists, von Willebrand factor’s-mediated adhesion, and the stabilization and growth of platelet thrombi. This allows for the assessment of the activity of both single and combined anti-platelet compounds with different modes of action. However, a limitation of this method is its inability to provide insight into the individual platelet activation pathways. For this reason, to assess the mechanisms of action of new anti-platelet compounds, other methods should be used in addition to T-TAS [34,35].

### 3.2. Measurement of the Exposition of P-Selectin, the Active form of GPIIb/IIIa on the Surface of Platelets in Whole Blood and Platelet Microparticles Formation

**Figure 2:**
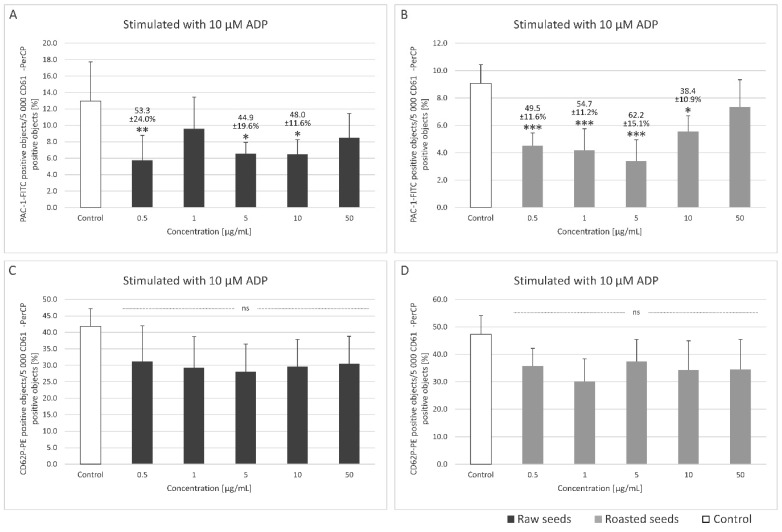
Effect of the extracts from raw and roasted sea buckthorn seeds (at concentrations of 0.5–50 μg/mL) on the exposition of the active form of GPIIb/IIIa on 10 µM ADP-stimulated blood platelets (**A**,**B**) and the exposition of P-selectin on 10 µM ADP-stimulated blood platelets (**C**,**D**) in whole blood. Blood platelets were gated based on their size and the exposition of CD61. In each sample, 5000 CD61-positive objects were acquired. To assess the exposition of GPIIb/IIIa, fluorescently conjugated monoclonal antibody PAC-1/FITC was used. Results are expressed as the percentage of platelets binding PAC-1/FITC. To assess the exposition of P-selectin, a fluorescently conjugated monoclonal antibody CD62P/PE was used. Results are expressed as the percentage of platelets binding CD62P/PE. Data represent the means ± SD. The blood samples were drawn from 5–6 healthy volunteers. The activity of the tested extract was compared to the control sample. The results were considered significant at *p* < 0.05 (* *p* < 0.05, ** *p* < 0.01, *** *p* < 0.001); ns—not significant.

**Figure 3:**
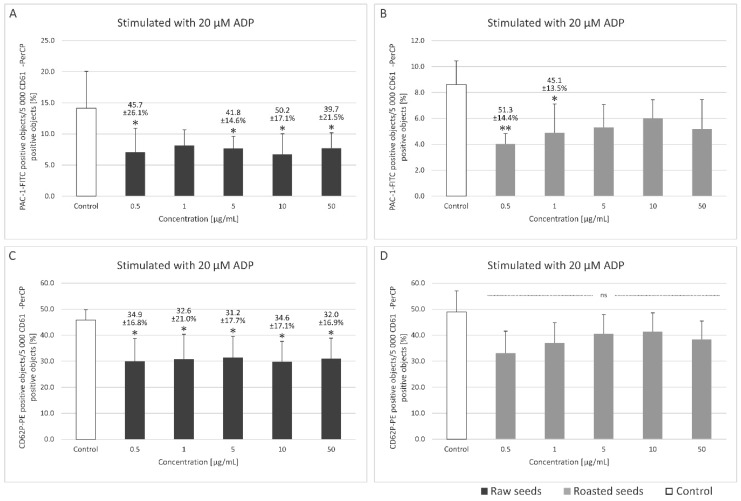
Effect of the extracts from raw and roasted sea buckthorn seeds (at concentrations of 0.5–50 μg/mL) on the exposition of the active form of GPIIb/IIIa on 20 µM ADP-stimulated blood platelets (**A**,**B**), and the exposition of P-selectin on 20 µM ADP-stimulated blood platelets (**C**,**D**) in whole blood. Blood platelets were gated based on their size and the exposition of CD61. For each sample, 5000 CD61-positive objects were acquired. To assess the exposition of GPIIb/IIIa, a fluorescently conjugated monoclonal antibody PAC-1/FITC was used. Results are expressed as the percentage of platelets binding PAC-1/FITC. To assess the exposition of P-selectin, fluorescently conjugated monoclonal antibody CD62P/PE was used. Results are expressed as the percentage of platelets binding CD62P/PE. Data represent the means ± SD. The blood samples were drawn from 5–6 healthy volunteers. The activity of the tested extract was compared to the control samples. The results were considered significant at *p* < 0.05 (* *p* < 0.05, ** *p* < 0.01); ns—not significant.

Figure 2 and Figure 3 show the markers of blood platelet activation (induced by 10 and 20 µM ADP, respectively) measured by flow cytometry. Changes in blood platelet activation were observed in whole blood treated with the two extracts at all tested concentrations (0.5–50 µg/mL), although they were not always statistically significant (Figure 2 and Figure 3). The extracts from both raw (at concentrations of 0.5, 5, and 10 μg/mL) and roasted (at concentrations of 0.5–10 μg/mL) sea buckthorn seeds inhibited the exposition of the active form of GPIIb/IIIa on the surface of platelets stimulated with 10 μM ADP by 38.4–62.2% (Figure 2A,B). In platelets stimulated by 20 μM ADP, significant inhibition (by 31.2–34.9%) of the exposition of P-selectin was observed for all the used concentrations of the extract from raw sea buckthorn seeds (0.5–50 µg/mL) (Figure 3C). The extract from raw (at concentrations of 0.5, 5, 10, and 50 μg/mL) and roasted (at concentrations of 0.5 and 1 μg/mL) seeds inhibited the exposition of the active form of GPIIb/IIIa on the surface of platelets stimulated with 20 μM ADP by 39.7–51.3% (Figure 3A,B).

Neither of the extracts at any of the used concentrations (0.5–50 µg/mL) significantly impacted platelet-derived microparticle formation or the exposition of P-selectin and the active form of GPIIb/IIIa on resting blood platelets and platelets activated by collagen (Supplementary Materials).

There are two main classes of ADP receptors on blood platelets: the P2X and P2Y receptors, each acting through different signaling cascades upon activation [36]. Shortly after exposure to ADP, GPIIb/IIIa receptor changes its conformation to a high-affinity state, which facilitates its binding to fibrinogen. PAC-1 monoclonal antibody allows for the detection of the high-affinity state GPIIb/IIIa, which is a marker of platelet activation [37]. Both sea buckthorn extracts could decrease the exposition of the active (high-affinity) form of GPIIb/IIIa on the surface of ADP-activated platelets, indicating anti-platelet activity. The extract from raw seeds also inhibited the exposition of P-selectin on the surface of platelets stimulated with 20 μg/mL ADP, although the effect was weaker than in the case of GPIIb/IIIa exposition. P-selectin is secreted from α granules upon platelet activation and is commonly used as a marker of these processes [36].

### 3.3. Measurement of Blood Platelet Adhesion to Collagen and Fibrinogen (in Washed Platelets)

**Figure 4:**
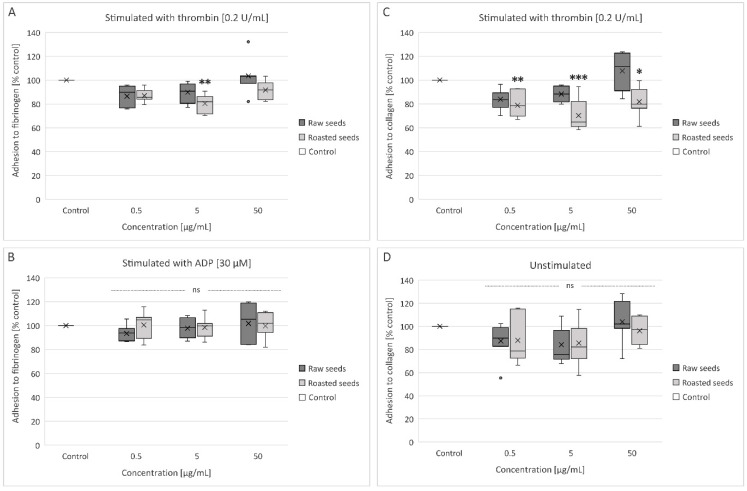
Effect of the extracts from raw and roasted sea buckthorn seeds (at concentrations of 0.5–50 μg/mL) on the adhesion of thrombin-activated platelets to fibrinogen (**A**), ADP-activated platelets to fibrinogen (**B**), thrombin-activated platelets to collagen (**C**), and the adhesion of unstimulated platelets to collagen (**D**) (*n* = 7). In the graphs, platelet adhesion is expressed as a percentage of the control sample (blood platelets without the tested extract). The data are expressed as medians and interquartile ranges. The results were considered significant at *p* < 0.05 (* *p* < 0.05, ** *p* < 0.01, *** *p* < 0.001); ns—not significant.

The anti-adhesive activity of the tested extracts was studied using washed human blood platelets; the results were presented as the percent of adhesion of control samples. As shown in Figure 4C, the level of adhesion of thrombin-activated blood platelets to collagen was reduced in the presence of all used concentrations of the roasted seeds extract: 0.5 μg/mL (by 21%, *p* = 0.069), 5 μg/mL (by 30%, *p* = 0.0002), and 50 μg/mL (by 21%, *p* = 0.0300). Moreover, the extract from roasted seeds at a concentration of 5 µg/mL inhibited the adhesion of thrombin-activated blood platelets to fibrinogen by approximately 20% (*p* = 0.0017) relative to control (Figure 4A). However, none of the tested concentrations of the extracts (0.5–50 µg/mL) demonstrated anti-adhesive properties when ADP-activated platelet adhesion to fibrinogen was measured (Figure 4B) and when unstimulated platelet adhesion to collagen was measured (Figure 4D).

It appears that the extract from roasted sea buckthorn seeds can only decrease the adhesion of thrombin-stimulated platelets. Thrombin is the strongest physiologically relevant platelet agonist [36]. PAR1 is the major thrombin receptor on human platelets; the GPIb-V-IX complex contributes to thrombin-induced platelet activation as well. Ligands that bind to the GPIb receptor have been shown to inhibit platelet activation by thrombin in vitro [38]. However, the exact mechanism of the anti-adhesive activity of sea buckthorn seed extract is not clear yet.

### 3.4. Measurement of Arachidonic Acid Metabolism and COX-1 Activity

**Figure 5:**
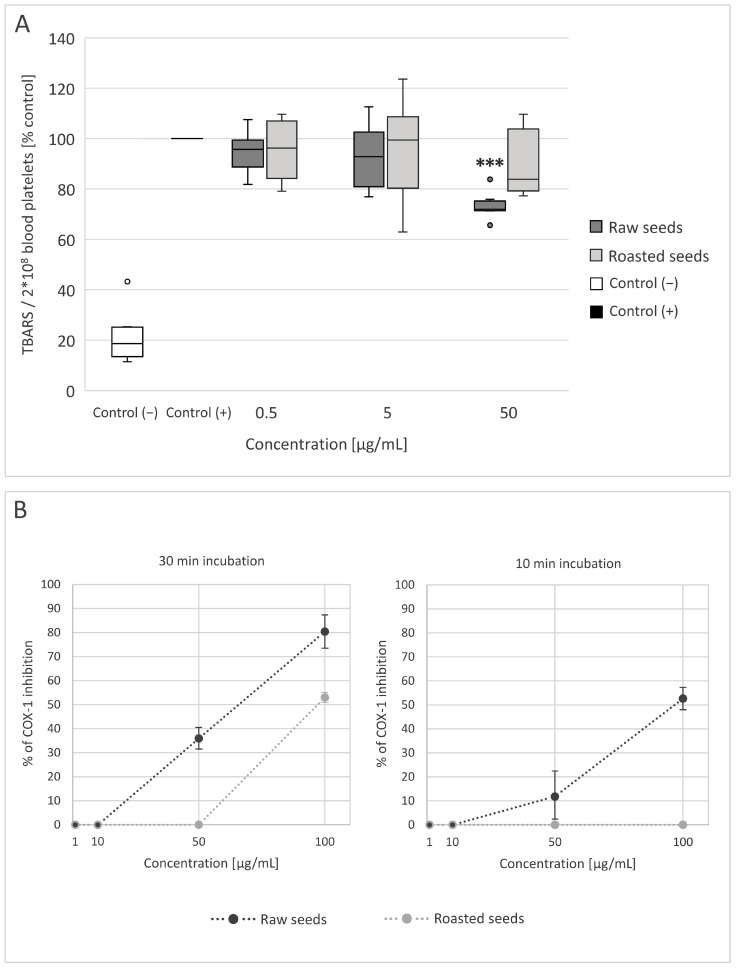
Effect of the extracts from raw and roasted sea buckthorn seeds (at concentrations of 0.5–50 μg/mL) on arachidonic acid metabolism in blood platelets stimulated by thrombin (5 U/mL) (*n* = 8) (**A**), and the activity of COX-1 (**B**). In (**A**), the results are presented as TBARS/2 × 10^8^ blood platelets (percent of the control sample). The data are expressed as medians and interquartile ranges. The results were considered significant at *p* < 0.05 (*** *p* < 0.001). The differences between negative and positive control were statistically significant (*p* < 0.001). In (**B**), the results are expressed as % of COX-1 inhibition (in comparison to 100% initial activity sample—sample where inhibitor vehicle (50% DMSO) was added instead of the extracts). The extracts were incubated with COX-1 for 10 or 30 min at the concentrations of 1, 10, 50, and 100 μg/mL. The data are shown as the means and SD of two dilutions of the sample (each measured in duplicate).

Blood platelet activation is associated with arachidonic acid metabolism, in which various intermediate products (including thromboxane A_2_) are produced [7]. In our experiment, TBARS concentration was used as an indicator of enzymatic peroxidation of arachidonic acid in platelets stimulated by thrombin. We observed that both extracts slightly reduced lipid peroxidation in blood platelets stimulated by thrombin, but only the extract from raw seeds at the concentration of 50 μg/mL caused a significant inhibition of TBARS levels, indicating an inhibition of arachidonic acid metabolism in blood platelets (*p* = 0.0004). The value was 28% lower in comparison to positive control (Figure 5A).

Cyclooxygenase is one of the key enzymes involved in arachidonic acid metabolism in blood platelets [39]. Several currently used clinical drugs that inhibit COX activity have severe side effects. For this reason, researchers are searching for new compounds that could achieve similar results but carry less risks. Plants are important sources of compounds used in drug discovery. According to Ambati et al. [40], 158 natural product inhibitors of COX were reported between years 2006 and 2019. They belong to many different classes, including flavonoids, alkaloids, coumarins, stilbenes, or anthraquinones [40,41].

Because the inhibition of arachidonic acid metabolism by the extracts could be linked to the inhibition of cyclooxygenase, we used a COX-1 inhibitor screening assay to determine if that is the case. Both extracts inhibited COX-1 activity after incubation with the enzyme for 30 min, although the extract from raw seeds showed stronger activity (approximately 93.5% inhibition at the concentration of 100 μg/mL, 35.9% inhibition at the concentration of 50 μg/mL, and no inhibition at the concentrations of 1 and 10 μg/mL) than the extract from roasted seeds (approximately 53% inhibition at the concentration of 100 μg/mL and no inhibition at the concentrations of 1–50 μg/mL). When the extracts were incubated with COX-1 for 10 min, the extract from raw seeds showed 52.6% (at 100 μg/mL) and 11.8% inhibition (at 50 μg/mL). The extract from raw seeds at the concentrations of 1–10 μg/mL and the extract from roasted seeds at all concentrations (1–100 μg/mL) did not inhibit COX-1 activity after 10 min of incubation (Figure 5B).

Our findings suggest that chemical compounds (including phenolic compounds) present in the extract from raw seeds may modulate blood platelet activation by interfering with arachidonic acid metabolism. Both extracts showed inhibitory activity, although the extract from raw seeds was stronger. This was consistent with the results of the arachidonic acid metabolism assay performed on blood platelets, where only the extract from raw seeds had a statistically significant effect, while the extract from roasted seeds lowered the level of TBARS slightly, but this change was not significant. These results indicate that the inhibition of COX-1 activity is one of the mechanisms of anti-platelet activity of the extracts from sea buckthorn seeds.

### 3.5. The Effect of the Extract from Raw and Roasted Sea Buckthorn Seed on Oxidative Stress in Plasma Treated with H_2_O_2_/Fe^2+^

None of the tested concentrations (5 and 50 µg/mL) of the extracts from raw and roasted seeds changed the level of 8-isoprostane in plasma treated with H_2_O_2_/Fe^2+^ (data not presented). Moreover, none of the tested concentrations (5 and 50 µg/mL) of the extracts changed the total non-enzymatic antioxidant status of plasma (data not presented).

In our previous study, we demonstrated that sea buckthorn seeds had antioxidant activity—they reduced lipid peroxidation, protein carbonylation, and thiol group oxidation in human plasma in vitro [12]. On the other hand, the extracts did not inhibit the oxidation of ABTS. This suggests that the mechanism responsible for their antioxidant properties might be linked to the activity of antioxidant enzymes. This seems to be supported by the results of Hua et al. [13], who studied the effect of an ethanolic extract from sea buckthorn seed residues on B16F10 (murine melanoma) cells treated with H_2_O_2_. The researchers observed an increase in the activity of catalase (CAT), superoxide dismutase (SOD), and glutathione peroxidase (GSH-Px). These results were further confirmed in an aging mice in vivo model.

### 3.6. A Comparison of the Anti-Platelet Activity of the Extract from Raw and Roasted Sea Buckthorn Seeds, and the Extract from Aronia Berries

The effects of the two used sea buckthorn seed extracts (5 µg/mL) on chosen blood platelet activities were compared with aronia berry extract (5 µg/mL) in Table 1. The aronia berry extract, which was used as a positive control (5 µg/mL), demonstrated anti-platelet properties. For example, it reduced the exposition of GPIIb/IIIa on blood platelets activated by 10 µM ADP and 20 µM ADP. Moreover, aronia berry extract had anti-adhesive properties. The extracts from sea buckthorn seeds demonstrated anti-platelet activity similar to aronia berry extract, reducing GPIIb/IIIa exposition on the surface of 10 and 20 µM ADP-activated blood platelets, P-selectin exposition on the surface of 20 µM ADP-activated blood platelets, and inhibiting the adhesion of thrombin-activated platelets to collagen. In addition, the anti-platelet properties (inhibition of adhesion of thrombin-activated platelets to collagen, inhibition of the exposition of the active form of GPIIb/IIIa on 10 and µM ADP-stimulated blood platelets, and inhibition of the exposition of P-selectin on 20 µM ADP-stimulated blood platelets) of sea buckthorn seeds were not significantly impaired by thermal processing (Table 1). On the other hand, only two effects were demonstrated by both types of seeds which are the inhibition of GPIIb/IIIa activation and no effect on P-selectin exposure in 10 µM ADP-stimulated platelets. All the other measured characteristics differ for two types of extracts from seeds, indicating that thermal processing can significantly change the anti-platelet properties (including anti-adhesive action) of the extract from raw sea buckthorn seeds (Table 1).

Many technologies utilized in the food industry are damaging to certain active compounds contained in fruits, vegetables, and seeds; food processing can significantly alter their biological properties. Thermal processing, one of the main methods of food processing, has been used since the ancient times [42,43]. It has many benefits, including improvement of shelf life and oxidative stability, inactivation of microorganisms, and reduction in the content of antinutritional compounds [44,45,46,47,48]. Thermal processing can also increase the yield of seed oil [49]. High temperatures can increase or decrease the content of phytochemicals in seeds and change their chemical structure. The outcome depends on the type of seeds and the conditions of processing, most importantly the method, temperature, and time [17,44]. For example, Li et al. [42] observed that roasting increases the phenolic content of sorghum, while in Djansang seeds, the total phenolic content was decreased by both boiling and roasting. Heat-induced change in the chemical content and structure of phytochemicals can also improve their biological activity [17]. For example, controlled thermal processing of black seeds (*Nigella sativa*) significantly increased their anti-proliferative activity in mouse colon carcinoma (MC38) in vitro [50]. On the other hand, high temperatures can also lead to the degradation of bioactive compounds and loss of activity. For example, roasting *Vicia faba* L. beans at 150 °C for 10–20 min significantly decreased their antioxidant capacity [51]. In our previous study, we showed that both raw and roasted sea buckthorn seeds had significant antioxidant activity, which was measured in human plasma in vitro [12]. Here, we observed that both raw and thermally processed seeds had anti-platelet potential, which was demonstrated in several assays, in full blood and washed blood platelets. In addition, thermal processing can significantly change the anti-platelet properties (for example, anti-adhesive properties) of sea buckthorn seed extract. Thermal processing is a beneficial technique that can improve various qualities of food and significantly alter its phytochemical content and biological activity; for this reason, the effect of high temperatures on bioactive phytochemicals should continue to be investigated.

Low toxicity and adequate bioavailability are important characteristics of plant extracts that are to be used as supplements or drugs. Although plant-derived compounds are mostly seen as safe by the general public, there is a lack of comprehensive data about the cyto- and genotoxic properties of many polyphenols. Toxicity studies are crucial, as some plant-based compounds might have detrimental effects, especially at high doses [52]. Moreover, the bioavailability of phenolic compounds may depend on the food source and the presence of other active compounds. Chong et al. [53] reported that procyanidins and anthocyanidins have beneficial cardiovascular properties in animal and human studies. In addition, phenolic extracts (for example, Aronox, an anthocyanin-rich commercial product made from aronia berries, which was used as a control in this study) were more effective anti-platelet agents than pure phenolic compounds not only in vitro but also in vivo. These results may suggest that phenolic compounds can have synergistic actions. Our present findings regarding extracts from raw and roasted sea buckthorn seeds (rich in various phenolic compounds, including proanthocyanidins) are consistent with these studies. Interestingly, isolated phenolic compounds are often less effective anti-platelet factors than the combined polyphenols of berries and other fruits or food products [53,54].

Unfortunately, many polyphenols have poor bioavailability, which decreases their effectiveness in vivo [55]. Guo et al., 2017 indicate that after enzymatic digestion, the phenolic compounds were quite different from the extracts from sea buckthorn berries. More flavonoid aglycones were released, but less total phenolic compounds, phenolic acids, and flavonoid glycosides were detected. In addition, the antioxidant activity of these berries was significantly enhanced by digestion [56]. Baeza et al., 2017 also noted that different metabolites of phenolic compounds are stronger inhibitors of blood platelet activation than their precursors [57].

Studies have shown that thermal processing can improve the bioavailability of different phytochemicals, as high temperatures can release the compounds bound to the food matrix or trapped behind the cell wall [16]. In our study, we used a concentration range of 0.5–50 µg/mL, because these concentrations can be achieved in blood during supplementation with phenolic compounds [58].

## 4. Conclusions

The present study provides new information about the biological properties of sea buckthorn seeds, demonstrating their anti-platelet activity that was not impaired by thermal processing. The extract from roasted seeds (50 µg/mL) significantly prolonged the time of occlusion measured by T-TAS—the AUC_10_ value was decreased by approximately 18%. Both extracts decreased the exposition of the active form of GPIIb/IIIa on the surface of platelets activated with 10 μM ADP (by 38.4–62.2%) and 20 μM ADP (by 39.7–51.3%). Moreover, the extract from raw seeds decreased the exposition of P-selectin on the surface of platelets stimulated with 20 μM ADP (by 31.2–34.9%). The adhesion of platelets stimulated with 0.2 U/mL thrombin to fibrinogen and collagen was inhibited only by the extract from roasted sea buckthorn seeds (by 20–30%). Moreover, the extract from raw seeds inhibited the level of TBARS (an indicator of enzymatic peroxidation of arachidonic acid) in washed platelets stimulated with 5 U/mL thrombin; the activity of COX-1 was inhibited by both extracts, though the effect of the extract from raw seeds was stronger.

Our findings suggest that the inhibition of blood platelet activation by the extracts from both raw and roasted seeds may be linked to the inhibition of arachidonic acid metabolism and decreasing the exposition of receptors on the surface of platelets. Because of their ability to reduce blood platelet activation, sea buckthorn seeds hold promise as novel and valuable phytopharmacological agents (new drugs or supplements) that improve the health of cardiovascular system and reduce the risk of cardiovascular diseases. Moreover, it is possible to use sea buckthorn seeds as an additive to act as a natural dietary supplement and at the same time improve the palatability of bread. An interesting and important finding of our study is that sea buckthorn seed extracts, similar to well-known aronia berry extract, have anti-platelet properties. However, more details about the mechanisms of their anti-platelet activity remain unclear and require further studies not only in vitro but also in vivo.

## Figures and Tables

**Table 1:** A comparison of the anti-platelet activity of the extract from raw and roasted sea buckthorn seeds (5 µg/mL), and the extract from aronia berries (5 µg/mL) in washed blood platelets (measured by adhesion to adhesive proteins (collagen and fibrinogen)) and in whole blood (measured by T-TAS and flow cytometry).

	Sea Buckthorn Raw Seeds	Sea Buckthorn Roasted Seeds	Aronia Berries
Inhibition of adhesion of thrombin-activated platelet to collagen	No effect	Anti-platelet activity	Anti-platelet activity
Inhibition of adhesion of thrombin-activated platelet to fibrinogen	No effect	Anti-platelet activity	No effect
Inhibition of thrombus formation (measured by T-TAS)	No effect	Anti-platelet activity	No effect
Inhibition of the exposition of the active form of GPIIb/IIIa on 10 µM ADP—stimulated blood platelets	Anti-platelet activity	Anti-platelet activity	Anti-platelet activity
Inhibition of the exposition of the active form of GPIIb/IIIa on 20 µM ADP—stimulated blood platelets	Anti-platelet activity	No effect	Anti-platelet activity
Inhibition of the exposition of P-selectin on 10 µM ADP—stimulated blood platelets	No effect	No effect	No effect
Inhibition of the exposition of P-selectin on 20 µM ADP—stimulated blood platelets	Anti-platelet activity	No effect	Anti-platelet activity

## Data Availability

Dataset available on request from the authors.

## Supplementary Materials

The following supporting information can be downloaded at: https://www.mdpi.com/article/10.3390/foods13152400/s1, Figure S1: Effect of the extracts from raw and roasted sea buckthorn seeds (at concentrations of 0.5–50 μg/mL) on the amount of platelet microparticles (PMP) in whole blood. Figure S2: Effect of the extracts from raw and roasted sea buckthorn seeds (at concentrations of 0.5–50 μg/mL) on the exposition of the active form of GPIIb/IIIa and P-selectin on unstimulated blood platelets in whole blood. Figure S3: Effect of the extracts from raw and roasted sea buckthorn seeds (at concentrations of 0.5–50 μg/mL) on the exposition of the active form of GPIIb/IIIa and P-selectin on 10 μg/mL collagen-stimulated blood platelets in whole blood.

## Abbreviations

ABTS—2:2′-azino-bis(3-ethylbenzothiazoline-6-sulfonic acid; ADP—adenosine diphosphate; AUC—area under the curve; CAT—catalase; COX—cyclooxygenase; CVDs—cardiovascular diseases; GSH-Px—glutathione peroxidase; PGF_2α_—prostaglandin F_2_ alpha; PGH_2_—prostaglandin H_2_; SOD—superoxide dismutase; TBARS—thiobarbituric acid reactive substances; T-TAS—Total Thrombus-formation Analysis System.
