# Supplementary material for: Anti-Platelet Activity of Sea Buckthorn Seeds and Its Relationship with Thermal Processing
Source: Foods. 2024 Jul 29;13(15):2400. doi: 10.3390/foods13152400 (PMC11312268; doi:10.3390/foods13152400)
Supplement: Supplementary file 1 [file foods-13-02400-s001.zip › foods-3111829-supplementary.pdf]

Figure S1

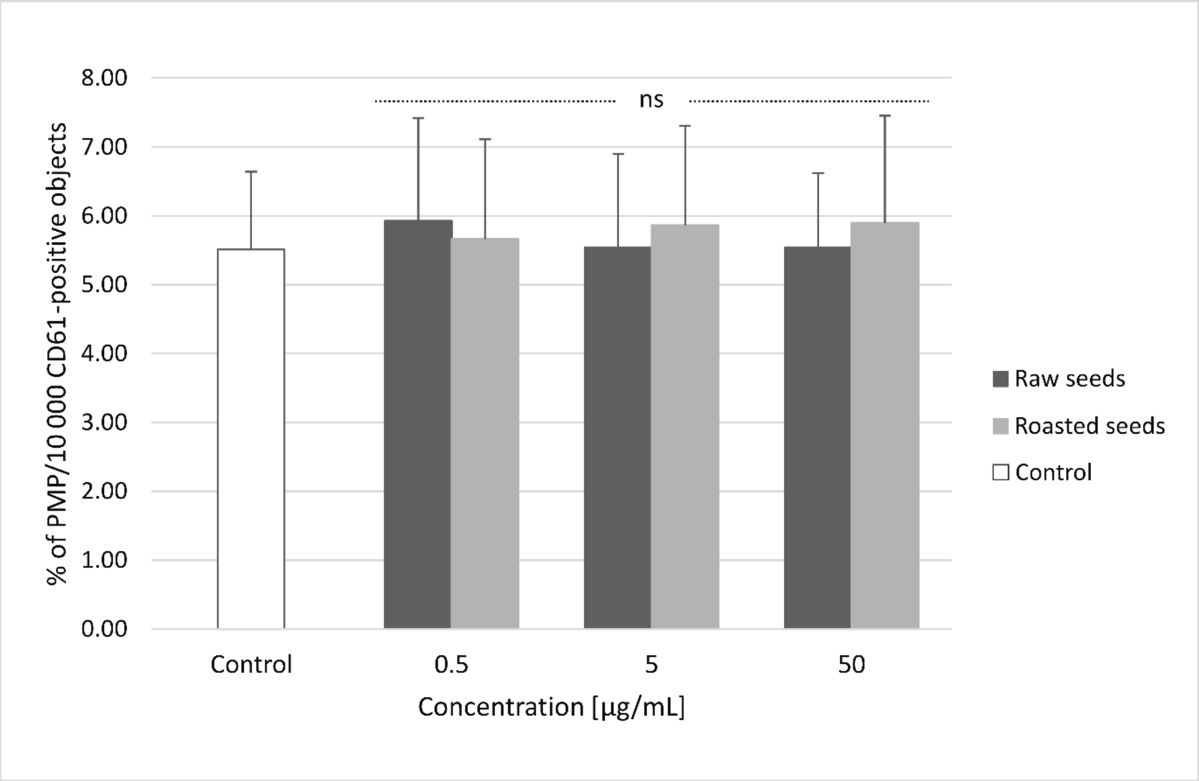

Figure S1. Effect of the extracts from raw and roasted sea buckthorn seeds (at concentrations of 0.5-50 µg/mL) on the amount of platelet microparticles (PMP) in whole blood. PMPs were gated based on their size and the exposition of CD61. For each sample, 10 000 CD61-positive objects were acquired. Results are expressed as the percentage of PMPs/10 000 CD61-positive objects. Data represent the means ± SD. The blood samples were drawn from 5 healthy volunteers. The activity of the tested extract was compared to the control samples. The results were considered as significant at $p<0.05$.

Figure S2A

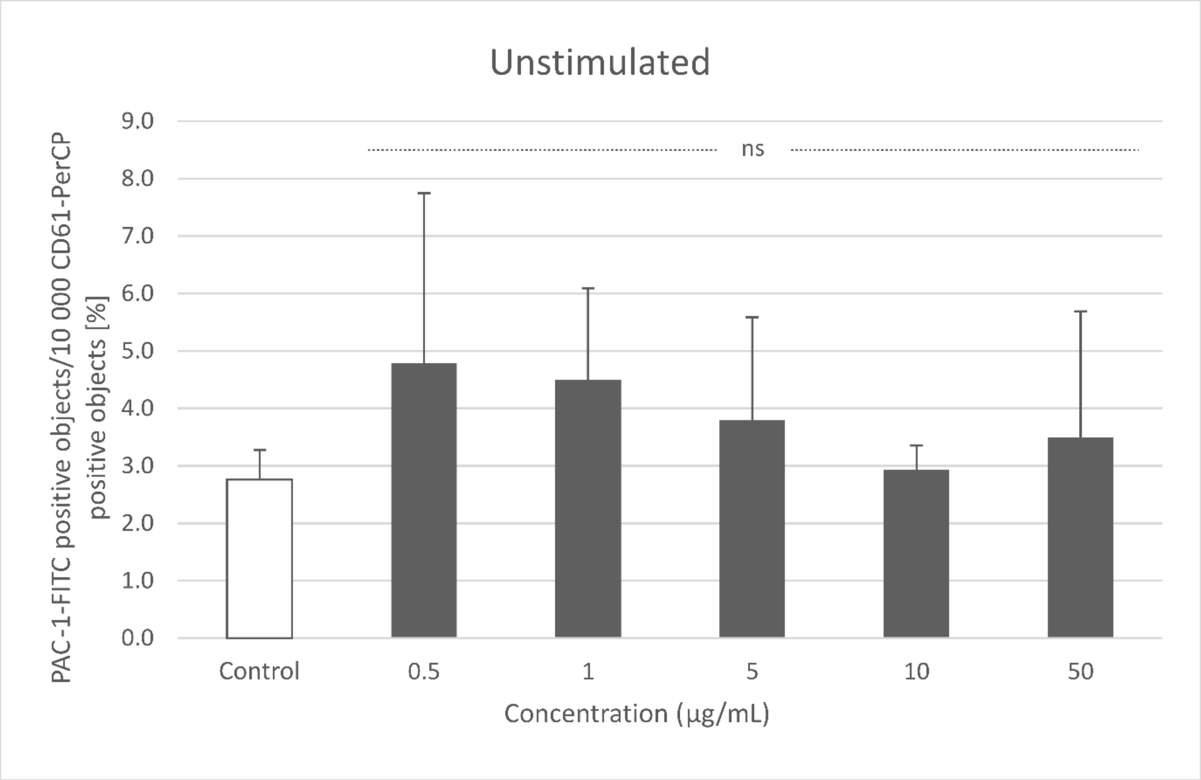

Figure S2B

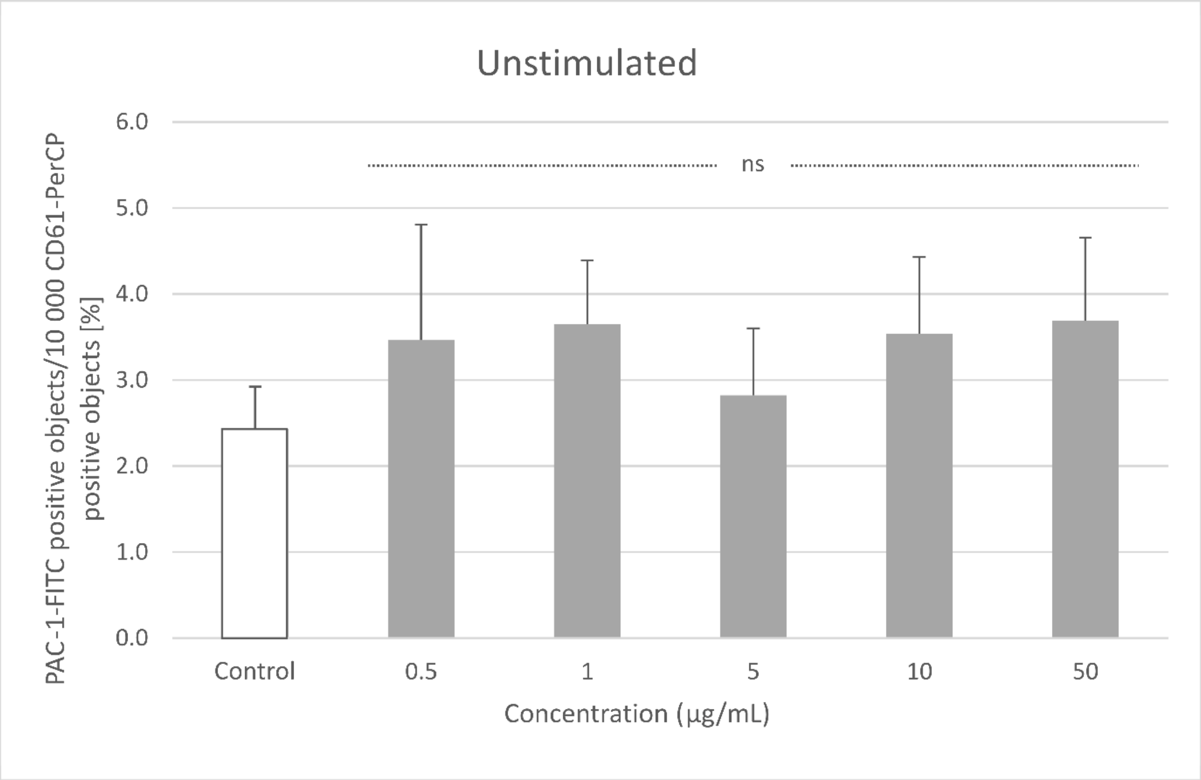

Figure S2C

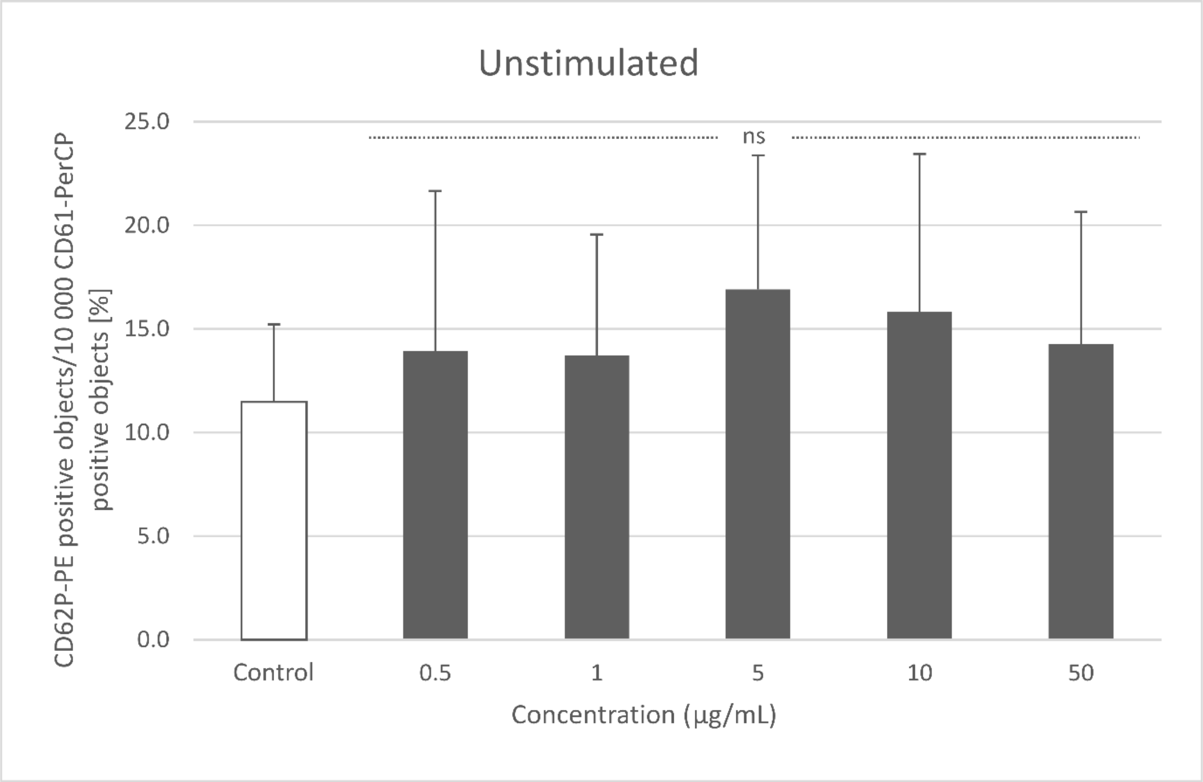

Figure S2D

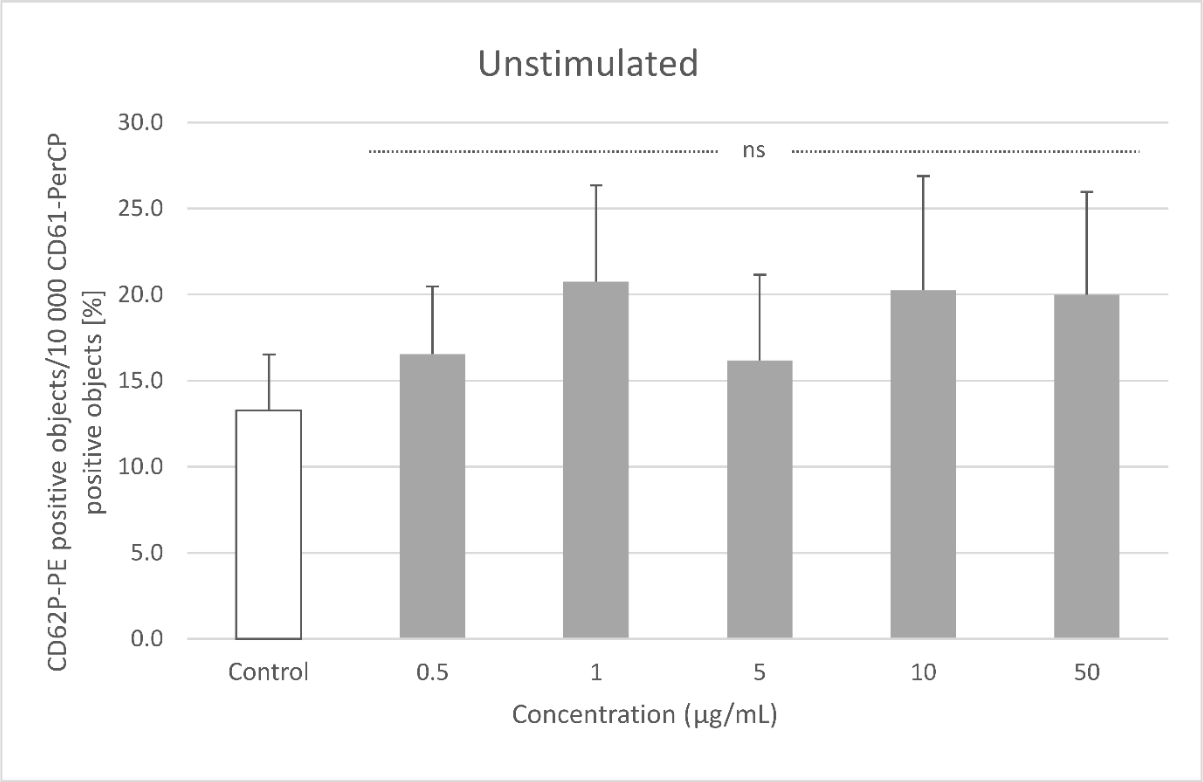

Figure S2. Effect of the extracts from raw and roasted sea buckthorn seeds (at concentrations of 0.5-50 µg/mL) on the exposition of the active form of GPIIb/IIIa (A and B) and P-selectin (B and D) on unstimulated blood platelets in whole blood. Blood platelets were gated based on their size and the exposition of CD61. For each sample, 5000 CD61-positive objects were acquired. To assess the exposition of GPIIb/IIIa, fluorescently conjugated monoclonal antibody PAC-1/FITC was used. Results are expressed as the percentage of platelets binding PAC-1/FITC. To assess the exposition of P-selectin, fluorescently conjugated monoclonal antibody CD62P/PE was used. Results are expressed as the percentage of platelets binding CD62P/PE. Data represent the means ± SD. The blood samples were drawn from 5-6 healthy volunteers. The activity of the tested extract was compared to the control samples. The results were considered as significant at $p<0.05$.

Figure S3A

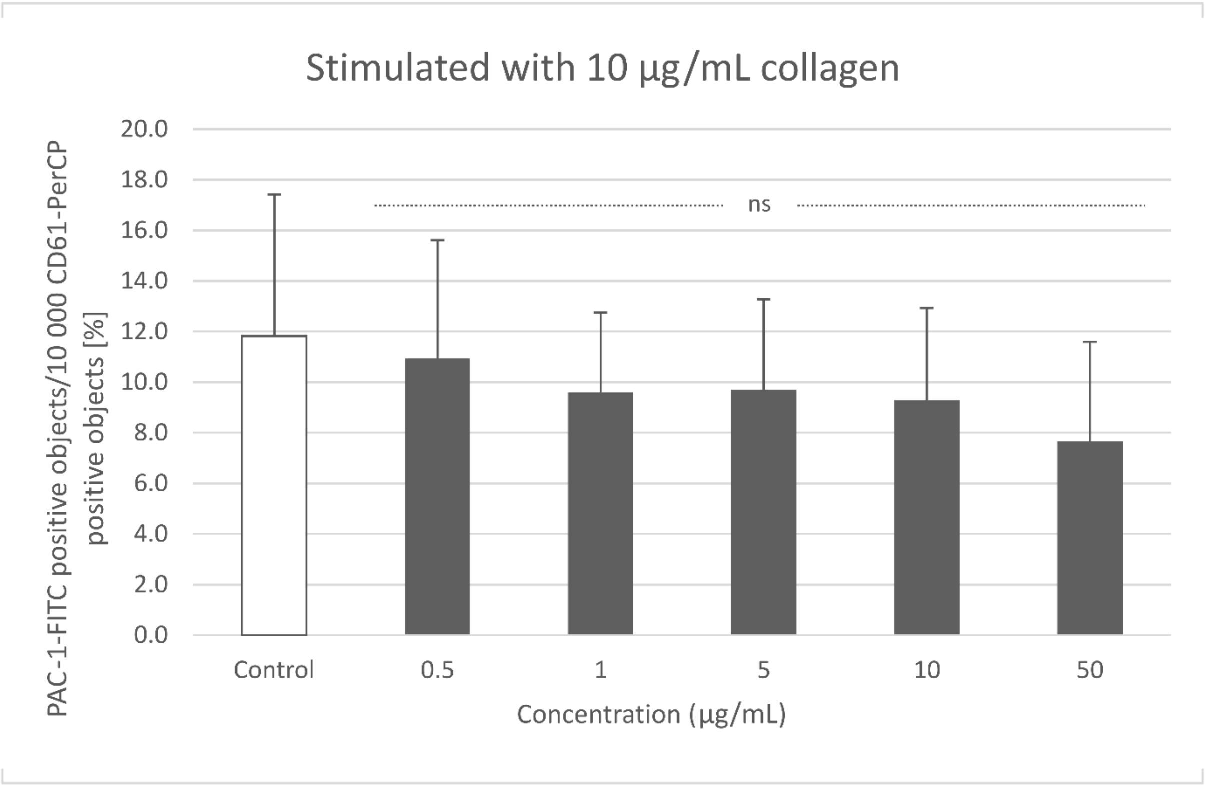

Figure S3B

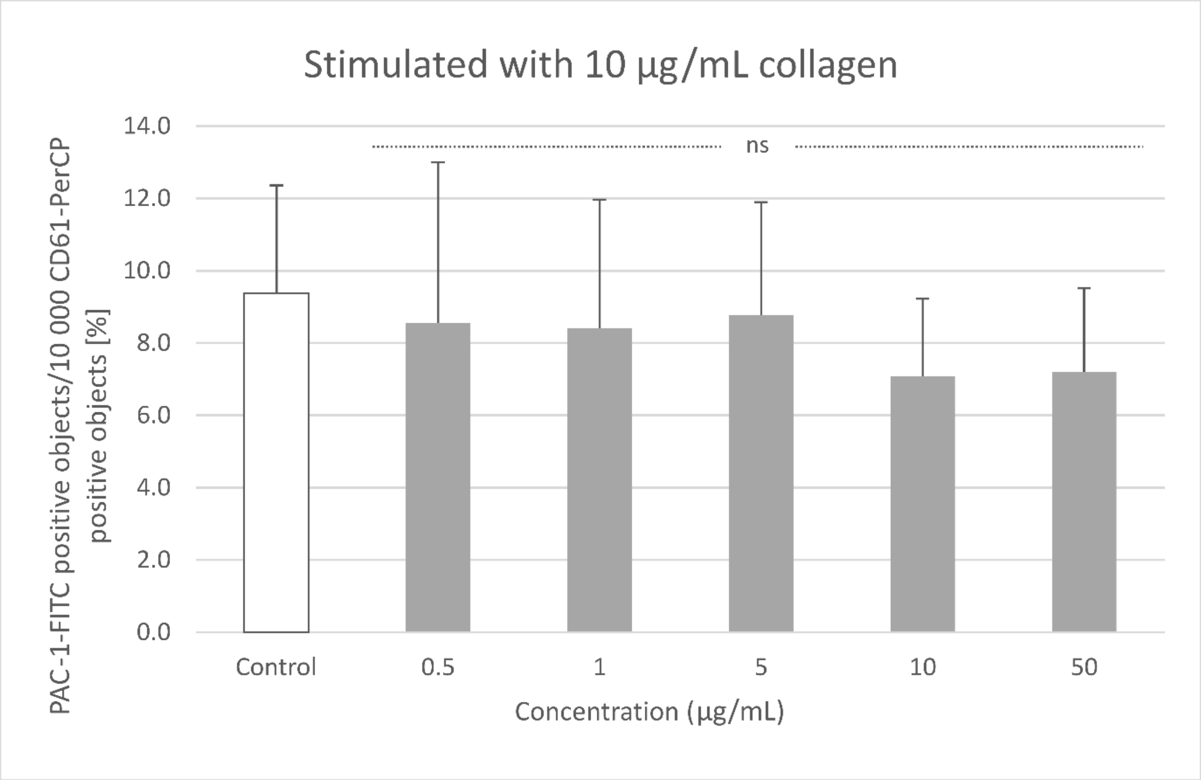

Figure S3C

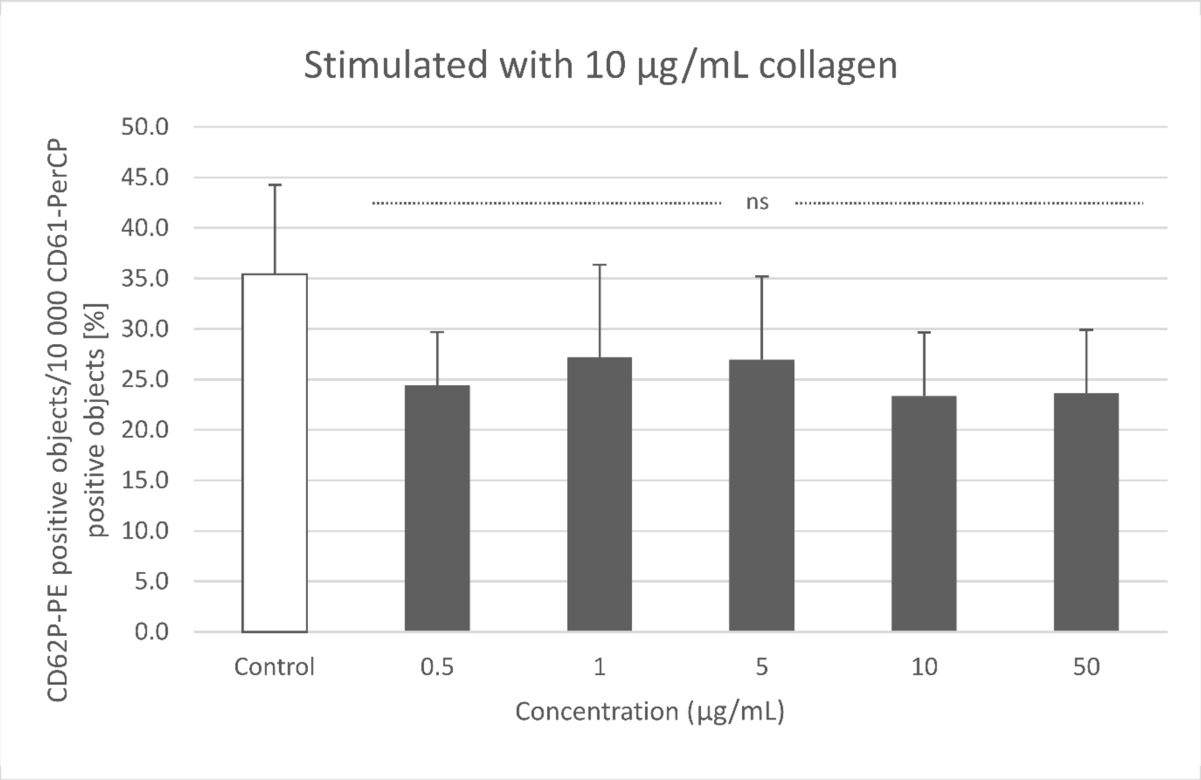

Figure S3D

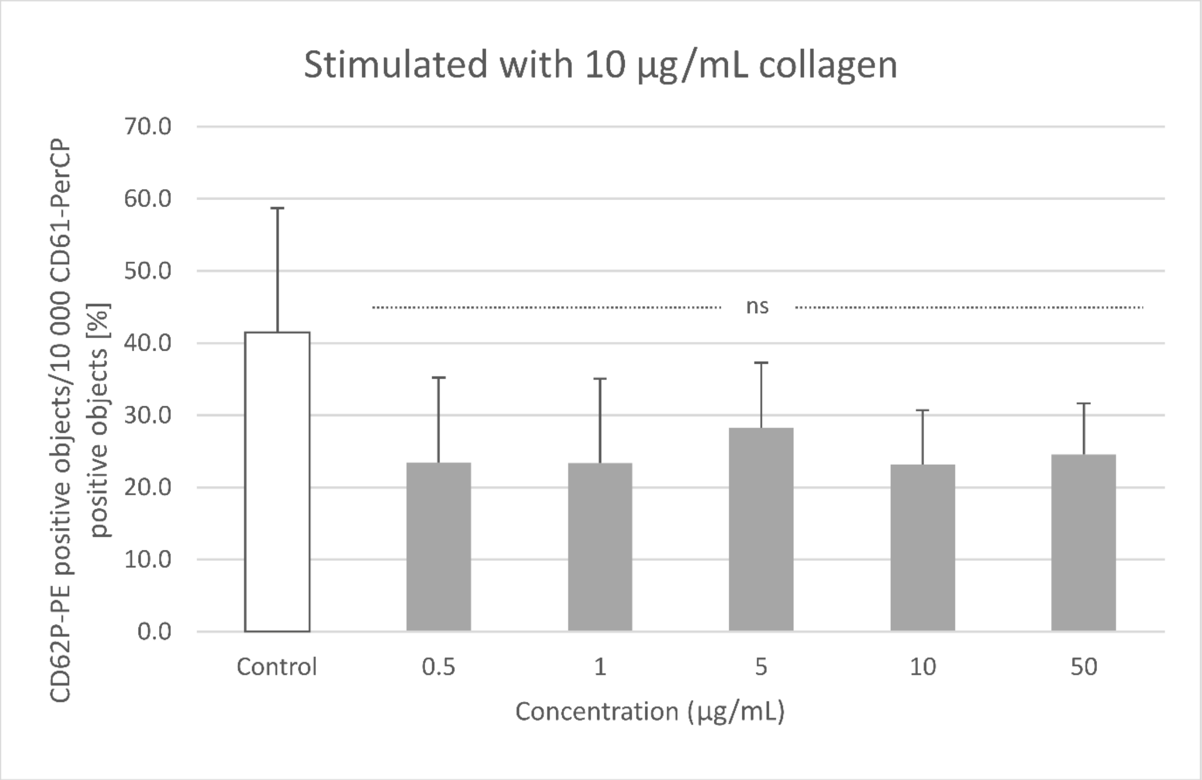

Figure S3. Effect of the extracts from raw and roasted sea buckthorn seeds (at concentrations of 0.5-50 µg/mL) on the exposition of the active form of GPIIb/IIIa (A and B) and P-selectin (B and D) on 10 µg/mL collagen-stimulated blood platelets in whole blood. Blood platelets were gated based on their size and the exposition of CD61. For each sample, 5000 CD61-positive objects were acquired. To assess the exposition of GPIIb/IIIa, fluorescently conjugated monoclonal antibody PAC-1/FITC was used. Results are expressed as the percentage of platelets binding PAC-1/FITC. To assess the exposition of P-selectin, fluorescently conjugated monoclonal antibody CD62P/PE was used. Results are expressed as the percentage of platelets binding CD62P/PE. Data represent the means ± SD. The blood samples were drawn from 5-6 healthy volunteers. The activity of the tested extract was compared to the control samples. The results were considered as significant at $p<0.05$.
